# Environmental Risk Assessment of Metals in the Volcanic Soil of Changbai Mountain

**DOI:** 10.3390/ijerph16112047

**Published:** 2019-06-10

**Authors:** Qing Ma, Lina Han, Jiquan Zhang, Yichen Zhang, Qiuling Lang, Fengxu Li, Aru Han, Yongbin Bao, Kaiwei Li, Si Alu

**Affiliations:** 1 Institute of Natural Disaster Research, Department of Environment, Northeast Normal University, Changchun 130024, China; maq706@nenu.edu.cn (Q.M.); hanln301@nenu.edu.cn (L.H.); lifx144@nenu.edu.cn (F.L.); arh690@nenu.edu.cn (A.H.); baoyb924@nenu.edu.cn (Y.B.); likw395@nenu.edu.cn (K.L.); als217@nenu.edu.cn (S.A.); 2 Jilin Institute of Geological Environment Monitoring, Changchun 130061, China; weifenfangcheng@tom.com; 3 Changchun Institute of Technology, Changchun 130021, China; 0215046@ccit.edu.cn; 4 Key Laboratory for Vegetation Ecology, Ministry of Education, Changchun 130117, China; 5 State Environmental Protection Key Laboratory of Wetland Ecology and Vegetation Restoration, Northeast Normal University, Changchun 130117, China

**Keywords:** Tianchi volcano, volcanic soil, metals, ecological risk assessment, human health risk assessment

## Abstract

Tianchi volcano is a dormant active volcano with a risk of re-eruption. Volcanic soil and volcanic ash samples were collected around the volcano and the concentrations of 21 metals (major and trace elements) were determined. The spatial distribution of the metals was obtained by inverse distance weight (IDW) interpolation. The metals’ sources were identified and their pollution levels were assessed to determine their potential ecological and human health risks. The metal concentrations were higher around Tianchi and at the north to the west of the study area. According to the geo-accumulation index (*I*_geo_), enrichment factor (EF) and contamination factor (CF) calculations, Zn pollution was high in the study area. Pearson’s correlation analysis and principal component analysis showed that with the exception of Fe, Mn and As, the metals that were investigated (Al, K, Ca, Na, Mg, Ti, Cu, Pb, Zn, Cr, Ni, Ba, Ga, Li, Co, Cd, Sn, Sr) were mostly naturally derived. A small proportion of Li, Pb and Zn may have come from vehicle traffic. There is no potential ecological risk and non-carcinogenic risk because of the low concentrations of the metals; however, it is necessary to pay attention to the carcinogenic risk of Cr and As in children.

## 1. Introduction

Volcanic activity is evidence of the dynamic behaviour of the earth’s interior and a manifestation of intense crustal movement [1]. Although volcanic eruptions are short-lived, the ash and pyroclastic material produced by volcanic eruptions can remain in the local environment for months, decades or even millions of years [2], and their effects can extend to hundreds of miles away from the erupting volcano [3]. In volcanic regions, pyroclastic materials have formed soils with unique physical, chemical and mineralogical properties, such as low bulk density, large water storage and high phosphate retention [4].

Soil, as the main component of biogeochemical system, is the source and sink of various types of pollutants [5]. It thus plays an important role in the storage and circulation of pollutants [6]. Pedological processes are influenced by many factors, including environmental conditions and parent rock properties [4]. Both human activities (e.g., the use of automobiles, fertilizers and pesticides, mining and metallurgical industries, wood processing) and geological activities (e.g., volcanic activities, earthquakes, landslides, debris flows) can introduce some major and trace elements into the environment [7]. The concentration of metals in the soil largely depends on the parent material [8]; thus, most metals accumulate after parent material decomposition and volcanic activity [9], resulting in high baseline concentrations of some metals in volcanic soil [10]. A large-scale volcanic eruption produces large amounts of ash and pyroclastic material, which contains not only nutrients (nitrogen, phosphorus, potassium, calcium, sodium, magnesium, etc.), but also toxic elements and metalloids (arsenic, mercury, etc.) [11].

Because metals are essentially non-biodegradable, they are environmentally persistent [12]. Excessive metals in the soil may pose certain levels of ecotoxicity and environmental hazard [13]. They may undergo many natural processes, such as dissolution, complexation, absorption and desorption, thus affecting the surrounding ecology and human health [14]. The toxicity of metals depends on the dose, exposure pathway, and duration of exposure [15]. Some trace elements (Zn, Cu, Mn) are essential nutrients for metabolism, but at high concentrations, these essential elements are also harmful to human health [16]. Metals in soil enter the body through three major exposure pathways: ingestion, inhalation and dermal contact [17], and may reach levels harmful to human health through the food chain [18,19]. Excess metal can biomagnify in human tissues; damage the nervous, kidney and reproductive systems [20]; act as an endocrine disruptor; disrupt the hormone system; and act as a carcinogen [21].

There are more than 200 large and small volcanoes in the Changbai Mountain area. Strong basalt eruptions occurred in the Pliocene, during which the total lava flow covered a total area of 15,000 km^2^ [22]. The Changbai volcanic field, consisting of Wangtian’e volcano (China), Tianchi volcano (China and North Korea), and Namphothe volcano (North Korea), represents the largest active volcanic system in the continental intraplate setting of north-east Asia [23]. Changbai Mountain is an important part of the Chang-Ji-Tu Pilot Zone, an area agglomerating tourism resources, ecological environment resources, forest specialty resources, hydropower resources and mineral resources [24], and is part of the world biosphere reserve network. More than 100,000 people live in urban and rural areas within 100 km of Tianchi. In the event of an eruption, it would cause great damage and loss to the ecology, personal safety and economy of the region. 

Experts and scholars have studied the volcanic formation mechanism, volcanic geological hazards, volcanic monitoring and warning, volcanic hazard risk and other aspects of Changbai Mountain [25,26,27]. However, there is still a lack of knowledge on the concentrations of metals and related risks in the volcanic soil and volcanic ash around Changbai Mountain. The main objectives of this research were to: (1) study the concentrations and distributions of metals in the volcanic soil and volcanic ash; (2) identify the sources of metals using statistical methods; (3) assess the ecological and human health risks of metals.

## 2. Materials and Methods

### 2.1. Study Area

**Figure 1:**
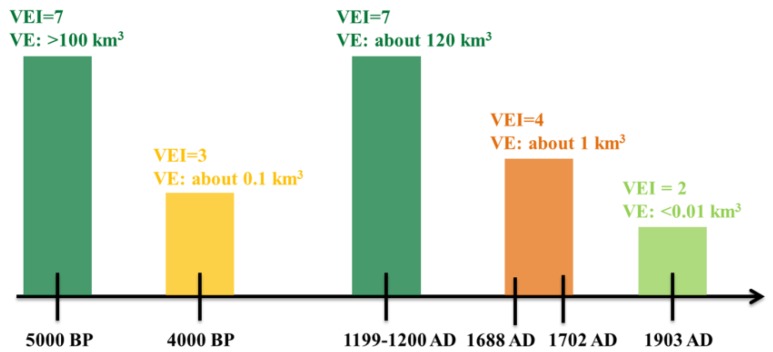
The number of eruptions, volume of eruption (VE) and volcanic explosive index (VEI) of Tianchi volcano.

Changbai Mountain is located in the south-east of Baishan City, Jilin Province. Tianchi volcano (41°35′–42°25′ N, 127°40′–128°16′ E) is the most well-preserved Cenozoic multi-genesis compound volcano in China. It is the source of the Songhua, Yalu and Tumen rivers [28]. There have been many eruptions in the recorded history of Tianchi volcano (5000 BP). Based on research by Liu [29], historical data, and the volcanic eruption scale table developed by Hong [22], we determined the number of eruptions, volume of eruptions (VE), and volcanic explosive index (VEI) of Tianchi volcano (Figure 1). The results show that Tianchi volcano has erupted at least five times [30]. Among these eruptions, the one that occurred about 1000 years ago, called the “Millennium eruption” by experts and scholars, was also one of the largest eruptions within the last 2000 years of human history [31]. In 2002–2005, Tianchi volcano displayed magmatic disturbance that gradually attracted extensive attention [32]. It is at the node of millennial and centennial resurrection periods, with potential eruption risk [33].

### 2.2. Sampling Collection and Analysis

**Figure 2:**
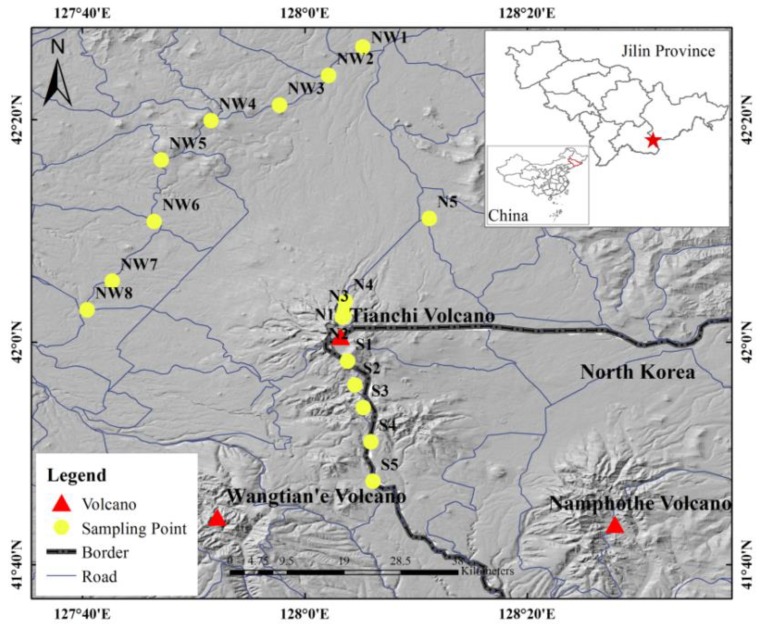
Distribution of sampling sites in Changbai Mountain.

In September 2018, samples were collected along the south, north and north-west slopes of Changbai Mountain, with Tianchi volcano at the centre and within a range of 60 km of the sampling sites. Samples were collected in accordance with the Technical Specification for Soil Environmental Monitoring (HJ/T 166-2004). A total of 29 samples were collected (25 volcanic soil samples (topsoil and subsoil) and 4 volcanic ash samples) at 18 sampling points (Figure 2). Samples were sealed and stored in labelled polyethylene bags and quickly transported to the laboratory for further treatment. The geographic coordinates of each sampling point were recorded using a global positioning system (GPS). Altitude, soil classification and vegetation type information of sampling points were extracted from geospatial data (SRTM 90 m, 1:1,000,000 Soil Maps of China, 1:1,000,000 Vegetation Atlas of China) (Table S1).

The samples were dried at room temperature for one week and then foreign materials such as gravel and plant roots were removed. The samples were ground with a porcelain mortar and then sieved through a 2 mm nylon mesh. Metal concentrations in samples were determined based on the National Standard Method of China (HJ 718-2016). An accurately weighed 0.5 g sample was placed in a 50 mL polytetrafluoroethylene crucible. Samples were digested with HCl–HNO_3_–HClO_4_–HF, and then filtered through a 0.45 μm filter membrane. The concentrations of 21 metals (Al, Fe, K, Ca, Na, Mg, Mn, Ti, Cu, Pb, Zn, Cr, Ni, Ba, Ga, Li, Co, Cd, As, Sn and Sr) were determined using inductively coupled plasma optical emission spectrometry (ICP-OES, Avio 200, PerkinElmer, MA, USA). The pH was determined in a soil:water (1:2.5) suspension using a pH meter (NY/Y 1377-2007). The organic matter content was determined by the K_2_Cr_2_O_7_-H_2_SO_4_ oxidation method, and the content of volcanic glass was determined by the hydrofluoric acid dissolution method [34]. Phosphate retention was determined according to the method in the book *Soil Survey Laboratory Methods*. Quality assurance and quality control were carried out by using certified reference materials (GSS series), analysing blank samples and repetitive analysis. Relative standard deviations between duplicate sets of data were less than 10%. 

### 2.3. Ecological Risk Assessment

In this study, soil geochemical background data refers to data from the China National Environmental Monitoring Centre (The Background Values of Soil Elements in Jilin Province) [35]. To assess the degree of metal contamination in volcanic soil and volcanic ash, the following indicators were calculated to assess the ecological risk of metals.

#### 2.3.1. Geo-Accumulation Index (I_geo_)

*I*_geo_ can be used to assess the degree of metal contamination in soil [36] and to determine which metals are enhanced in soil [37]. It is calculated using the following expression (Equation (1)):

(1) $$I_{\mathrm{geo}}{\;=\;\log}_{2}\left(C_{n}/{1.5B}_{n}\right)$$

where *C_n_* is the concentration of metals in soil; and *B_n_* is the geochemical background value in soil. The constant 1.5 is used to correct for fluctuations of metals in the background values due to natural processes [36]. *I*_geo_ values are classified into seven levels: unpolluted (≤0); unpolluted to moderately polluted (0–1); moderately polluted (1–2); moderately to highly polluted (2–3); highly polluted (3–4); highly to extremely highly polluted (4–5); extremely highly polluted (≥5). 

#### 2.3.2. Enrichment Factor (EF)

EF is used to assess the degree of metal enrichment in soil [38], and it is also an effective tool to distinguish between the sources of metals (natural or anthropogenic activities) [39,40]. Conservative elements such as Al, Fe, Mn, Ti and Ca are usually chosen as reference elements. The selected elements should have stable chemical properties, be present at high natural concentrations in the crust, and not be subject to interference by external factors [41]. Considering Ti is an important major element in soils and the least likely to be influenced by external factors, Ti was selected as a reference element in this study. EF was calculated by Equation (2):

(2) $$\mathrm{EF}\;=\;\frac{{\left(C_{M}/C_{Ti}\right)}_{S}}{{\left(C_{M}/C_{Ti}\right)}_{B}}$$

where (*C_M_/C_Ti_*)*_S_* and (*C_M_/C_Ti_*)*_B_* are the concentration ratios of metals to the reference element (Ti) in the sample and in the geochemical background value, respectively. An EF value of <1.5 indicates that the element originates from the crust or through the natural weathering process [42]. The enrichment is evaluated as follows: EF values of <1 indicate no enrichment; values of 1–3 indicate minor enrichment; values of 3–5 indicate moderate enrichment; values of 5–10 indicate moderately severe enrichment; values of 10–25 indicate severe enrichment; values of 25–30 indicate very severe enrichment and values >50 indicate extremely severe enrichment.

#### 2.3.3. Contamination Factor (CF)

CF is used to assess the level of contamination in soil, and was calculated by Equation (3):

(3) $$CF\;=\;\frac{C_{n}}{B_{n}}$$

According to Hakanson (1980) [43], *CF* values of <1 indicate low contamination; values of 1–3 indicate moderate contamination; values of 3–6 indicate considerable contamination and values of >6 indicate very high contamination.

#### 2.3.4. Potential Ecological Risk Index (RI)

The potential ecological risk index is an indicator of the ecological effects of metals on the environment, and comprehensively evaluates the potential ecological risk caused by metals according to their toxicity and environmental response [44]. RI was calculated by Equations (4) and (5):

(4) $${Er}^{i}{\;=\;Tr}^{i}{\times C}_{f}^{i}{=Tr}^{i}\times \frac{C_{s}^{i}}{C_{B}^{i}}\;$$

(5) $$\mathrm{RI}\;=\;\overset{n}{\underset{i=1}{\sum }}{Er}^{i}$$

where *Er^i^* is the potential ecological risk factors for a given element i; $C_{f}^{i}$ is the contamination factor; and *Tr^i^* is the toxic response factor. Hakanson (1980) [43] reported the toxicity coefficients of seven heavy metals. Xu [45] recalculated the toxicity coefficients of 12 metals (Zn, Cu, Ni, Pb, V, Co, Cr, As, Cd, Hg, Ti and Mn) according to Hakanson’s calculation principle. In this study, 9 toxicity coefficients were selected to evaluate their potential risks (As = 10, Cu = Pb = Ni = Co = 5, Cr = 2, Zn = Ti = Mn = 1). Hakanson’s classification standard for RI was based on 8 pollutants. We analyzed 9 metals; therefore, the classification standard should be adjusted according to the number and type of metals [46]. We recalculated the RI classification according to Wang [46] and Chen [47], and the results are shown in Table S2.

### 2.4. Health Risk Assessment

The United States Environmental Protection Agency (USEPA) health risk assessment model was adopted to evaluate the hazards of metals in the volcanic field soil. There are three main pathways of metal intake: ingestion, inhalation and dermal contact [48]. The average daily dose (ADD) of each metal was calculated using the parameters in Table S3 and with the following Equations (6)–(8):

(6) $${\mathrm{ADD}}_{\mathrm{ing}}\;=\;\frac{C\times \mathrm{IngR}\times \mathrm{EF}\times \mathrm{ED}}{\mathrm{BW}\times \mathrm{AT}}{\times 10}^{-6}$$

(7) $${\mathrm{ADD}}_{\mathrm{inh}}\;=\;\frac{C\times \mathrm{InhR}\times \mathrm{EF}\times \mathrm{ED}}{\mathrm{PEF}\times \mathrm{BW}\times \mathrm{AT}}$$

(8) $${\mathrm{ADD}}_{\mathrm{dermal}}\;=\;\frac{{C\times \mathrm{SA}\times \mathrm{SAF}\times \mathrm{ABS}}_{d}\times \mathrm{EF}\times \mathrm{ED}}{\mathrm{BW}\times \mathrm{AT}}{\times 10}^{-6}$$

The non-carcinogenic risk of metals was calculated by Equations (9) and (10):

(9) $$\mathrm{HQ}\;=\frac{\mathrm{ADD}}{\mathrm{RfD}}$$

(10) $$\mathrm{HI}\;=\;\overset{n}{\underset{i=1}{\sum }}\mathrm{HQ}$$

where hazard quotient (*HQ*) is a non-carcinogenic risk of a single metal; *RfD* is the reference dose of metals in mg·kg^−1^·day^−1^ (Table S3); hazard index (HI) is the total non-carcinogenic risk of metals in three exposure routes. Values of HI >1 indicate that non-carcinogenic risk may occur, while HI values <1 indicate no significant risk. 

Cr, Ni and As are considered carcinogenic elements, and their potential carcinogenic risks were calculated by the following Equations (11) and (12):

(11) CR = ADD×SF

(12) $$\mathrm{TCR}\;=\overset{n}{\underset{i=1}{\sum }}\mathrm{CR}$$

where *CR* is the carcinogenic risk of a single metal; SF is the cancer slope factor of metals (Table S3); and TCR is the total carcinogenic risk of Cr, Ni and As in the three exposure routes. If *CR* ≤ 1 × 10^−6^, then the risk is considered to be negligible; if 1 × 10^−6^ < CR < 1 × 10^−4^, then the risk is acceptable; if *CR* ≥1 × 10^−4^, then the metal poses a potential carcinogenic risk.

### 2.5. Data Treatment

The metal concentrations and the risk values were calculated by Excel 2010, and the figures were drawn using Origin 8.0 software (OriginLab, Hampton, MA, USA). The Pearson’s correlation analysis and principal component analysis (PCA) were performed using SPSS 24 software (IBM, Armonk, NY, USA). The concentrations of different metals in volcanic soil and volcanic ash were compared by one-way analysis of variance using Minitab 17 software (State College, PA, USA). Inverse distance weight (IDW) is a commonly used spatial interpolation method, which takes the distance between interpolation points and sample points as the weight to carry out weighted average calculations. This method is simple, intuitive and efficient. IDW interpolation can predict the metal concentration value at an unknown point; the software output is in the form of a graph of the metal concentration distribution, thus evaluating the metal pollution in the region, and accurately locating the highly polluted regions [47]. We used IDW interpolation to predict metal concentrations using ArcGIS 10.2 software (Esri, RedLands, CA, USA). 

## 3. Results and Discussion

### 3.1. Soil Properties

The properties of volcanic soil samples are summarized in Table 1. The pH ranged from 4.51–5.84, with a mean of 5.16, making it a partially acidic soil. The organic matter ranged from 1.25% to 15.69%, with a mean value of 8.45%. Because the samples were taken from forest soil, the organic matter content was high [49]. Because organic matter has a high affinity for metals [50], these results indicate that the soil has a strong ability to accumulate metals [51]. Volcanic glass and phosphate retention are important indicators of volcanic soil properties [52]. Volcanic glass content ranged from 66.38–88.26%, with a mean value of 78.26%; the high volcanic glass content is proof of the volcanic nature of the soil. The phosphate retention ranged from 9.47% to 48.99%, with a mean value of 33.50%; 80% of the samples had values greater than 25%, which meets the standard of volcanic soils [53]. Therefore, both volcanic glass and phosphate retention properties of the samples in the study area show the volcanic nature of the soil.

**Figure 3:**
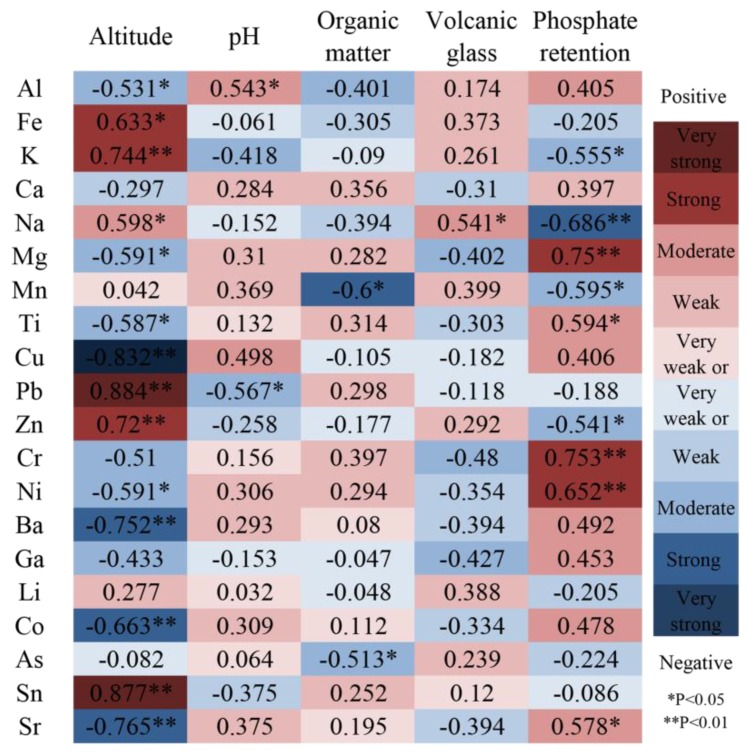
Pearson’s correlation coefficient between metal concentration and altitude, pH, organic matter, volcanic glass and phosphate retention.

The correlations between metal concentration and altitude, pH, organic matter, volcanic glass and phosphate retention (Figure 3) were determined using Pearson’s correlation analysis. Cu, Pb and Sn were very strongly correlated (|r^2^| = 0.8–1) and Co, Sr, Ba, Zn, Fe and K were strongly correlated (|r^2^| = 0.6–0.8) with altitude. Both pH and volcanic glass were weakly or very weakly correlated with 16 metals; and organic matter was moderately correlated (|r^2^| = 0.4–0.6) with 17 metals. Cr, Ni, Mg and Na were strongly correlated with phosphate retention. Generally speaking, there were strong correlations between metal concentrations and altitude and phosphate retention, and most of the metals were significantly correlated (*p* < 0.05). 

### 3.2. Metal Concentrations and Distributions

**Figure 4:**
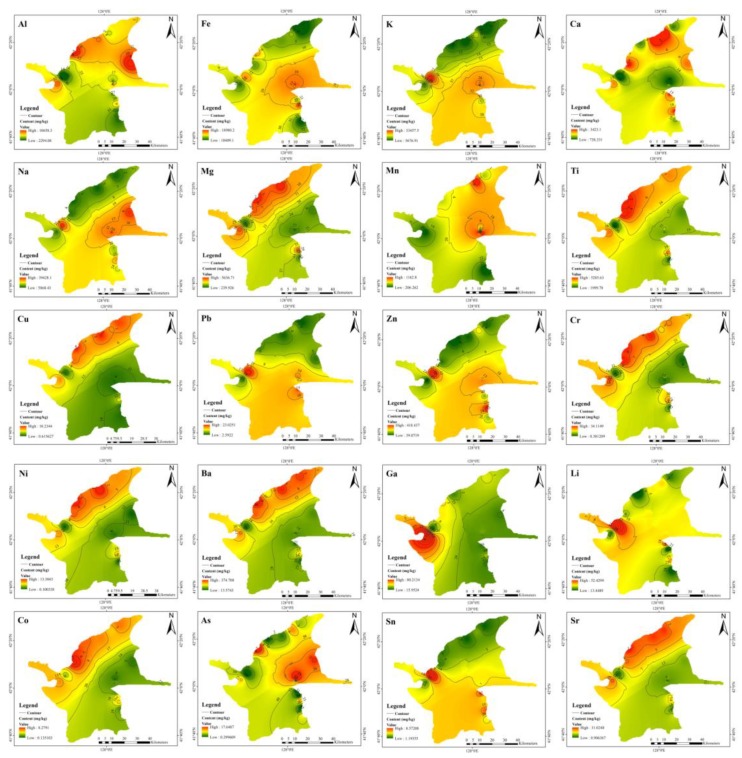
The spatial distribution of the 20 metals in Changbai Mountain.

The concentrations of metals in the volcanic soil and volcanic ash are shown in Figure 4 and Table 2. Cd was not detected in any soil samples, indicating that the concentration of Cd in soil was low, which may be related to the affinity of Cd with organic matter. It has been reported that the presence of organic matter can reduce the amount of Cd adsorbed to soil [54]. Figure 4 shows the spatial distribution of the metal concentrations, and the contour map for each metal clearly indicates their “fingerprints” in volcanic soil and volcanic ash [20]. In the colour coding, green and red represent lower and higher concentrations of each metal, respectively. Fe, K, Na, Mn, Pb, Zn, As and Sn had similar distributions, i.e., their concentrations were higher near Tianchi. Ca, Mg, Ti, Cu, Cr, Ni, Ba, Co and Sr also had similar distributions; the concentrations of these metals were higher in the north to the west of the study area. Table 2 shows the mean, standard deviation (SD) and coefficient of variation (CV) of metal concentrations in volcanic soil and volcanic ash. The SD is highly correlated with the average concentration of metals, and the CV reflects their distribution. The SDs of Al, Fe, K, Na, Mg and Ti were large; and the metals were mainly derived from parent materials of the soil, indicating that these six metals were mainly derived from highly heterogeneous environments [55,56]. The CVs ranged from 6.16% to 174.19%; within this range, the CVs of Na, Mg, Cu, Pb, Zn, Cr, Ni, Ba, Co, As, Sn and Sr were greater than 50%, indicating that these metals are distributed variably or non-homogeneously in space [51]. 

One-way ANOVA was used to assess the differences in metal concentrations in different sample types (volcanic soil and volcanic ash) (Table 2). The *F* value was much larger than 1, indicating that the difference between the mean values of each group was statistically significant. The results show that the concentrations of K, Na, Mg, Ti, Cu, Zn, Cr, Ni, Ba, Ga, Li, Sn and Sr differed significantly between different sample types (*p* < 0.05), indicating that the concentrations of these 13 metals were related to the sample types. However, sample types had no significant effects on the concentrations of Al, Fe, Ca, Mn, Pb, Co and As (*p* > 0.05).

The average concentrations of most metals were lower than previously reported background values (Table S4); and only K, Na, Ti, Zn, Ga, Li, Sn exceeded the background values. Among these, the content of Zn in volcanic soil and volcanic ash exceeds the background value by 1.02 and 3.56 times, respectively, indicating that the level of Zn pollution in the study area was high. As the new standard (GB 15618-2018) only applies to agricultural land, we have used the original standard for our study area, a non-agricultural land. Based on the Environmental quality standard for soils (GB 15618-1995), the average concentrations of As, Cu, Pb, Cr and Ni were less than Grade Ι levels. The Changbai Mountain Nature Reserve, established in 1960, is one of the earliest and most important protected areas in China. It was declared a National Nature Reserve in 1986. Therefore, the pollution in Changbai is low, and the metals mainly derive from volcanoes and the soil parent material. However, the concentration of Zn in the volcanic soil and volcanic ash were relatively high for this area. A majority (68.97%) of the samples had Zn concentrations that exceed the Grade Ι limit (100 mg·kg^−1^), and 20.69% of the samples contained Zn in excess of the Grade II limit (300 mg·kg^−1^). Large volcanic complexes are all characterised by geogenic enrichment of Zn [57]. In addition, higher Zn concentration may also be derived from anthropogenic activities. Changbai Mountain is a nationally famous scenic spot, attracting increasing numbers of tourists, and increased traffic flows. Under these conditions, Zn in automobile exhaust, tires, engines, fuels, and lubricants will be discharged into the environment [58,59], resulting in Zn pollution in soil. 

The average concentrations of metals in the volcanic soil and volcanic ash from Tianchi volcano and other volcanoes around the world are summarized in Table S4. The concentrations of major elements (Al, Fe, K, Ca, Na, Mg, Ti), except for Na and Ti, were much lower in Tianchi volcano than in other volcanoes (except Cordón Caulle of Chile). The average concentrations of Mn (except for Cordón Caulle of Chile), Cu, Cr (except for Vesuvius of Italy), Ni, Ba (except for Wusu Tianshan of China), Co, Cd and Sr in Tianchi volcano were lower than those in other volcanoes. However, the average concentrations of Zn, Ga and Sn in Tianchi volcano were higher than those in other volcanoes. Overall, the average concentrations of most metals in Tianchi volcano were either lower or similar to those of other volcanoes. The concentrations of only a few metals (such as Zn) were very high at Tianchi volcano. The differences in metal concentrations among volcanic samples may be due to their specific environmental characteristics, such as anthropogenic inputs and topographic features [55]. 

### 3.3. Source Identification

**Figure 5:**
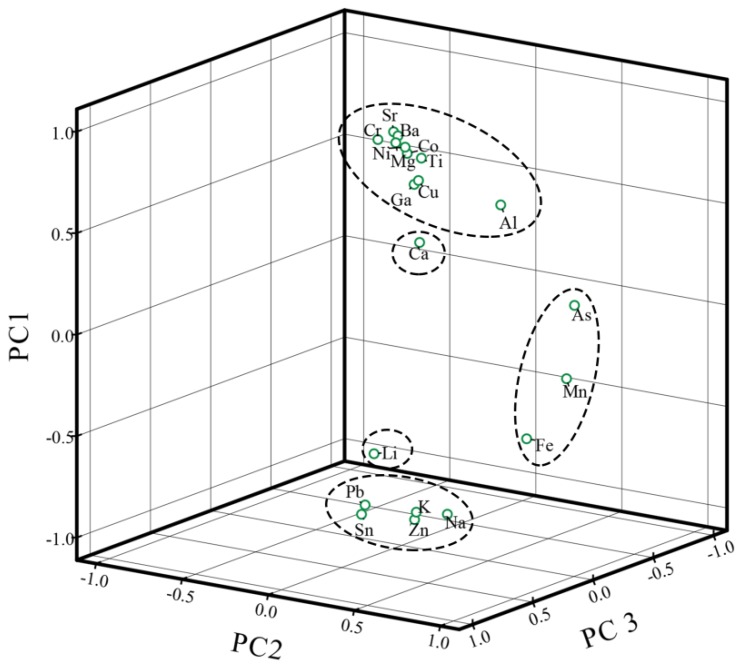
Principal component analysis of 20 metals in volcanic soil and volcanic ash.

Pearson’s correlation analysis and principal component analysis (PCA) were used to identify the relationships between and sources of metals in volcanic soil and volcanic ash (Table S5 and Figure 5). Correlation analysis (Table S5) shows strong correlations (0.6 < coefficients < 0.8) between many metals. In general, high correlations between elements indicate that they may have similar sources [60]. As shared significant and moderately strong correlations with Mn and Fe (0.4 < coefficients < 0.6, *p* < 0.05); similarly, Mn shared significant and moderately strong correlations with As and Fe. Therefore, As, Mn and Fe may share a common source. Ca had no strong correlation with any metal, while Li had a moderate correlation with Pb and strong correlation with Zn.

Prior to PCA, the Kaiser–Meyer–Olkin (KMO) measure and Bartlett’s test of sphericity were performed on the data. The KMO value is 0.724, which is larger than the recommended cut-off threshold of 0.5 [61], and the *p-*value is 0.000 < 0.05, indicating that the data is suitable for PCA. Four principal components with eigenvalues greater than 1 were extracted; these accounted for 83.82% of the cumulative total variance. PC1 had high factor loadings for Al, K, Na, Mg, Ti, Cu, Pb, Zn, Cr, Ni, Ba, Ga, Co, Sn and Sr, which accounted for 59.61% of the total variance. PC2 (Fe, As and Mn), PC3 (Li) and PC4 (Ca) accounted for 59.61%, 11.52% and 7.59% of the total variance, respectively.

The results of Pearson’s correlation analysis and PCA were consistent, proving the reliability of the results. In general, Al, K, Ca, Na, Mg and Ti have been reported to be abundant in soil and are not affected by human activities [62]. Therefore, PC1 and PC4 are likely to share similar, mainly natural, sources. Although Fe is abundant in the crust, the content of Fe in soil was very vulnerable to external factors because iron products have been widely used in people’s lives. As may be derived from atmospheric deposition [63]. Although Mn may be derived from crystals, it may also come from automobile exhaust. In order to improve the octane number of gasoline, some manganese-containing antiknock agent is added to gasoline, thus increasing the Mn content in the environment and causing environmental pollution [9]. We consider PC2 to mainly reflect anthropogenic sources. Pearson’s correlation analysis shows a strong correlation between Li and Pb and Zn. Although Pb and Zn are derived mainly from natural sources, because the content of Zn was much higher than the background value, these levels may reflect the fact that Zn is widely used in automotive manufacturing and the mechanical industry. Similarly, Pb is the main component of automobile exhaust [64], Li is often used in lubricants of automobile parts, as well as in lithium battery vehicles, etc. Therefore, it is likely that Li, Zn and Pb have both natural and anthropogenic sources. 

### 3.4. Contamination and Ecological Risk Assessment

**Figure 6:**
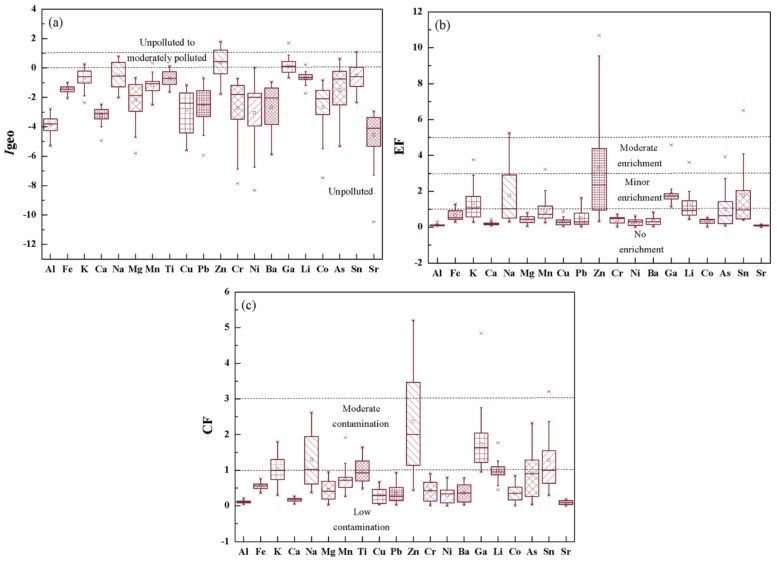
Box-plots of (**a**) geo-accumulation (*I*_geo_), (**b**) enrichment factor (EF) and (**c**) contamination factor (CF).

The values of EF, *I*_geo_ and CF were calculated to quantify soil pollution (Figure 6). *I*_geo_ and EF were used to evaluate the enhancement above background levels. The *I*_geo_ values of most metals, on average, were less than 0, which is similar to the background, indicating unpolluted levels. The average values of Na, Zn and Ga were between 0 and 1, indicating unpolluted to moderately polluted levels. The EF value can provide preliminary information on the sources of metals in volcanic soil and volcanic ash. The EF values of most metals were less than 1, indicating no enrichment. The average EF values of K, Na, Ga, Li, As and Sn were between 1 and 3, indicating minor enrichment. Only the average EF value of Zn was between 3 and 5, indicating moderate enrichment. EF values less than 1.5 indicate that the natural sources of metals may be dominant; and EF values greater than 1.5 indicate that there is enrichment corresponding to anthropogenic input. Among the samples, 37.9%, 41.4%, 10.3%, 10.3%, 58.6%, 93.1%, 24.1%, 24.1% and 44.8% had EF values greater than 1.5 for K, Na, Mn, Pb, Zn, Ga, Li, As and Sn respectively, indicating that these metals had both natural and anthropogenic sources. The CF value was used to assess the pollution level of metals. The average CF value for each metal yielded the following order: Zn (2.37) > Ga (1.74) > Na (1.29) > Sn (1.28) > K (1.02) > 1. CF values between 1 and 3 indicate moderate contamination, and values below 1 indicate low contamination. Overall, the *I*_geo_, EF and CF results show that the contents of metals in volcanic soils and volcanic ash in the study area were low, and there was almost no enrichment and pollution of metals, except for Zn. 

**Figure 7:**
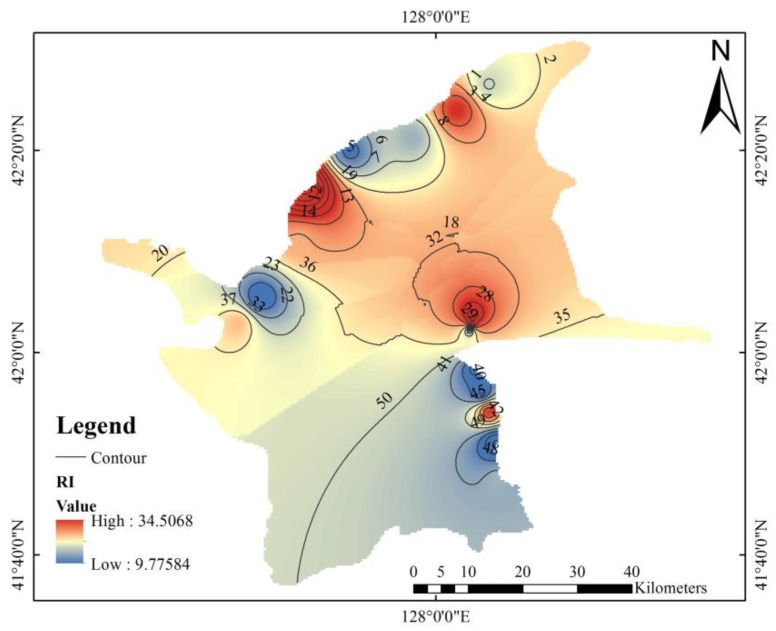
Spatial distribution of potential ecological risk index (RI) in Changbai Mountain.

High levels of some metals may increase the potential toxicity risk to the ecosystem. The RI values were calculated to assess the potential ecological risk of nine metals and determine the adverse ecological effects of metals (Figure 7). The comprehensively determined RI values of nine metals in the study area ranged from 9.77 to 34.51, with an average value of 19.65. These values are less than 40, which is considered an indicator of slight ecological risk. RI was relatively high around Tianchi and near the road from the North Slope to the West Slope. Because the ecological toxicity of each element is different, the results of total metal concentration and comprehensive ecological risk may not agree [65]. Overall, the potential ecological risk posed by the metals in the study area is low, and the ecological risk to the existing biological communities is small. We do not expect any adverse biological effects due to metal pollution. 

### 3.5. Human Health Risk Assessment

Table 3 lists the calculated values for the non-carcinogenic risks of metals in volcanic soil and volcanic ash to children and adults. For children, non-carcinogenic risk values of all metals, in descending order, are as follows: ingestion (5.80 × 10^−1^) > inhalation (2.77 × 10^−2^) > dermal (1.21 × 10^−2^). Ingestion was the main exposure route, accounting for 93.58% of HI. For adults, non-carcinogenic risk of all metals, in descending order, are as follows: dermal (3.72 × 10^−1^) > ingestion (6.21 × 10^−2^) > inhalation (6.81 × 10^−3^). Dermal was the main exposure route, accounting for 84.37% of HI. The total HQ values of the three metal exposure routes were, in descending order, as follows: As > Mn > Cr > Pb > Zn > Ni > Co > Cu for children and Cr > As > Mn > Pb > Zn > Ni > Co > Cu for adults. Their orders were basically similar, with As, Mn, Cr and Pb constituting the main metals that pose non-carcinogenic risk. Although children had HI values greater than adults, both groups had HI values below 1, indicating no potential non-carcinogenic risk in the study area. 

Table 4 lists the calculated values of the carcinogenic risks (CR) of metals in volcanic soil and volcanic ash to children and adults. Because not all metals have SF reference values, only the carcinogenic risks of Cr, Ni and As were calculated. For inhalation, the CR to both children and adults was less than 1 × 10^−6^, indicating that the risk is negligible. However, the CR value of Cr and As via ingestion was more than 1 × 10^−4^ for children, indicating potential carcinogenic risk. The TCR for adults posed by Cr, Ni, As was 6.24 × 10^−5^, indicating that the cancer risk was acceptable. However, the TCR for children posed by Cr, Ni, As was 3.36 × 10^−4^, exceeding the maximum acceptable risk level, and thus indicating potential carcinogenic risk. 

Overall, Cr and As have very high non-carcinogenic and carcinogenic risk values, which may affect the health of children and adults, and should be viewed with more attention. Children are at higher risk of exposure than adults, which may be due to their hand-to-mouth activity, their developing bodies, and their poor ability to detoxify and excrete toxins [66]. Therefore, metal exposure poses a higher risk for children, which should arouse widespread concern in society. Relevant government agencies should propose effective measures to protect children’s health.

## 5. Conclusions

The levels of volcanic glass and phosphate retention in the samples were relatively high, which is consistent with the properties of volcanic soil. The concentrations of Fe, K, Na, Mn, Pb, Zn, As and Sn were high near Tianchi, and the concentrations of Ca, Mg, Ti, Cu, Cr, Ni, Ba, Co and Sr were high at the west to the north of the study area. Levels of metals in volcanic soil and volcanic ash in the study area were considered safe, but the concentrations of Zn were higher, specifically, 1.02 and 3.56 times higher than the background values, respectively. The average concentrations of Na, Ti, Zn, Ga and Sn in the volcanic soil and volcanic ash of Tianchi volcano were higher than those of volcanoes in other parts of the world. Aside from Fe, Mn and As, which may be derived from human activities, metals in volcanic soil and volcanic ash in the study area mainly come from natural sources. However, Pb, Zn and Li may be partly derived from anthropogenic sources. The ecological risk of metals in the study area is low, however, attention should be paid to the health risks caused by Cr and As, especially their carcinogenic risk to children. Overall, pollution control in the study area has been good, but attention should be paid to the enrichment of Mn, Pb, Zn, and Li from vehicle traffic caused by the development of tourism. This is especially true for the enrichment of Zn. In addition, since children are more vulnerable to the risks posed by metals, it is necessary to pay attention to the impact of metals on children’s health in the volcanic area and propose effective measures to protect children’s health.

## Figures and Tables

**Table 1:** Properties of volcanic soil samples.

	pH	Organic Matter (%)	Volcanic Glass (%)	Phosphate Retention (%)
Min	4.51	1.25	66.38	9.47
Max	5.84	15.69	88.26	48.99
Mean	5.16	8.45	78.26	33.50

**Table 2:** Standard deviation (SD), coefficient of variance (CV) and one-way analysis of variance results of metal concentrations in volcanic soil and volcanic ash.

Metal	Volcanic Soil			Volcanic Ash			*F*	*p*
	Concentration (mg·kg^−1^)	SD	CV (%)	Concentration (mg·kg^−1^)	SD	CV (%)		
Al	6967	2350	33.73	5465	1006	18.41	1.16	0.291
Fe	14,939	2873	19.23	15,973	984	6.16	0.5	0.488
K	18,663	7229	38.73	27,470	7759	28.25	5.03	0.033 *
Ca	2201	712	32.35	1892	874	46.19	0.61	0.44
Na	17,497	10910	62.35	34,968	3946	11.28	9.79	0.004 **
Mg	3498	1786	51.06	497.1	122.9	24.72	10.94	0.003 **
Mn	451.8	216.8	47.99	518.6	184.4	35.56	0.34	0.566
Ti	4230	1324	31.30	2161	242	11.20	9.44	0.005 **
Cu	5.781	3.348	57.91	0.95	0.296	31.16	8.07	0.008 **
Pb	9.43	6.77	71.79	16.16	7.16	44.31	3.36	0.078
Zn	162.2	94.5	58.26	367	59.3	16.16	17.37	<0.001 **
Cr	22.63	12.59	55.63	0.824	0.702	85.19	11.64	0.002 **
Ni	7.285	4.378	60.10	0.1498	0.1291	86.18	10.3	0.003 **
Ba	221.7	130.9	59.04	24.32	15.28	62.83	8.81	0.006 **
Ga	30.72	12.77	41.57	17.143	1.521	8.87	1.38	0.046 *
Li	27.77	7.7	27.73	40.66	11.48	28.23	8.51	0.007 **
Co	4.585	2.959	64.54	1.946	0.622	31.96	3.07	0.091
As	8.04	5.87	73.01	3.1	5.4	174.19	2.49	0.126
Sn	2.933	1.863	63.52	6.76	1.844	27.28	14.59	0.001 **
Sr	19.97	11.06	55.38	3.72	2.39	64.25	8.33	0.008 **

* Significant at 0.05 level, ** Significant at 0.01 level.

**Table 3:** Non-carcinogenic health risk from metals in the volcanic soil and volcanic ash.

Element	Exposure Pathways	*RfD*	HQ	
			Children	Adult
Mn	Ingestion	4.60 × 10^−2^	1.28 × 10^−1^	1.37 × 10^−2^
	Inhalation	1.43 × 10^−5^	1.15 × 10^−2^	6.49 × 10^−3^
	Dermal	1.40 × 10^−1^	2.40 × 10^−4^	3.23 × 10^−3^
Cu	Ingestion	4.00 × 10^−2^	1.64 × 10^−3^	1.75 × 10^−4^
	Inhalation	4.02 × 10^−2^	4.55 × 10^−8^	2.56 × 10^−8^
	Dermal	1.20 × 10^−2^	3.11 × 10^−5^	4.18 × 10^−4^
Pb	Ingestion	3.50 × 10^−3^	3.79 × 10^−2^	4.06 × 10^−3^
	Inhalation	3.52 × 10^−3^	1.05 × 10^−6^	5.93 × 10^−7^
	Dermal	5.24 × 10^−4^	1.44 × 10^−3^	1.94 × 10^−2^
Zn	Ingestion	3.00 × 10^−1^	8.12 × 10^−3^	8.69 × 10^−4^
	Inhalation	3.00 × 10^−1^	2.27 × 10^−7^	1.28 × 10^−7^
	Dermal	6.00 × 10^−2^	2.31 × 10^−4^	3.11 × 10^−3^
Cr	Ingestion	3.00 × 10^−3^	8.36 × 10^−2^	8.96 × 10^−3^
	Inhalation	2.86 × 10^−5^	2.45 × 10^−4^	1.38 × 10^−4^
	Dermal	6.00 × 10^−5^	2.38 × 10^−2^	3.20 × 10^−1^
Ni	Ingestion	2.00 × 10^−2^	4.03 × 10^−3^	4.32 × 10^−4^
	Inhalation	2.06 × 10^−2^	1.09 × 10^−7^	6.16 × 10^−8^
	Dermal	5.40 × 10^−3^	8.50 × 10^−5^	1.14 × 10^−3^
Co	Ingestion	2.00 × 10^−2^	2.70 × 10^−3^	2.89 × 10^−4^
	Inhalation	5.71 × 10^−6^	2.64 × 10^−4^	1.49 × 10^−4^
	Dermal	1.60 × 10^−2^	1.92 × 10^−5^	2.59 × 10^−4^
As	Ingestion	3.00 × 10^−4^	3.14 × 10^−1^	3.36 × 10^−2^
	Inhalation	5.00 × 10^−5^	5.26 × 10^−5^	2.96 × 10^−5^
	Dermal	3.00 × 10^−4^	1.79 × 10^−3^	2.40 × 10^−2^
HI			6.19 × 10^−1^	4.41 × 10^−1^

*RfD* is the reference dose of metals in mg·kg^-1^·day^-1^. HQ is hazard quotient.

**Table 4:** Carcinogenic health risk from metals in the volcanic soil and volcanic ash.

Element	Exposure Pathways	SF	CR	
			Children	Adult
Cr	Ingestion	0.5	1.25 × 10^−4^	1.34 × 10^−5^
	Inhalation	42	2.94 × 10^−7^	1.66 × 10^−7^
	Dermal	-	-	-
Ni	Ingestion	0.84	6.77 × 10^−5^	7.25 × 10^−6^
	Inhalation	0.84	1.89 × 10^−9^	1.07 × 10^−9^
	Dermal	-	-	-
As	Ingestion	1.5	1.41 × 10^−4^	1.51 × 10^−5^
	Inhalation	15.1	3.97 × 10^−8^	2.24 × 10^−8^
	Dermal	3.66	1.96 × 10^−6^	2.64 × 10^−5^
TCR			3.36 × 10^−4^	6.24 × 10^−5^

CR is the carcinogenic risk of a single metal; SF is the cancer slope factor of metals.

## Supplementary Materials

The following are available online at https://www.mdpi.com/1660-4601/16/11/2047/s1, Table S1: Description and characteristics of the sampling sites, Table S2: The classification of potential ecological risk, Table S3: Parameter values used in health assessment, Table S4: Tianchi volcano and global distribution of metals in volcanic soil and volcanic ash, Table S5: Pearson’s correlation coefficient between metals in volcanic soil and volcanic ash.
