# Supplementary material for: Environmental Risk Assessment of Metals in the Volcanic Soil of Changbai Mountain
Source: Int J Environ Res Public Health. 2019 Jun 10;16(11):2047. doi: 10.3390/ijerph16112047 (PMC6604000; doi:10.3390/ijerph16112047)
Supplement: Supplementary file 1 [file ijerph-16-02047-s001.pdf]

Table S1. Description and characteristics of the sampling sites.

Site ID	Location name	Latitude	Longitude	Altitude (m)	Soil classification	Vegetation type
N1	North Slope	42°02'14.5896"N	128°03'24.2748"E	1967	Andosols	Alpine tundra
N2	North Slope	42°02'20.3964"N	128°03'31.3776"E	1934	Andosols	Alpine tundra
N3	North Slope	42°02'39.2424"N	128°03'33.5304"E	1869	Andosols	Alpine tundra
N4	North Slope	42°03'38.9700"N	128°03'38.448"E	1753	Cambisols	Alpine tundra
N5	North Slope	42°11'6.8460"N	128°11'12.1416"E	1127	Alfisols	Cold temperate and temperate mountain coniferous forests
NW1	North-West Slope	42°26'31.4484"N	128°5'14.1108"E	712	Alfisols	Temperate deciduous broad-leaved forest
NW2	North-West Slope	42°23'58.56"N	128°02'8.9448"E	751	Alfisols	Temperate deciduous broad-leaved forest
NW3	North-West Slope	42°21'18.6588"N	127°57'43.5816"E	873	Alfisols	Temperate deciduous broad-leaved forest
NW4	North-West Slope	42°19'54.1321"N	127°51'34.1170"E	1057	Alfisols	Temperate deciduous broad-leaved forest
NW5	North-West Slope	42°16'21.4464"N	127°47'5.3700"E	1088	Alfisols	Mixed forest of temperate coniferous and deciduous broadleaf
NW6	North-West Slope	42°10'50.6784"N	127°46'27.7320"E	928	Alfisols	Temperate deciduous broad-leaved forest
NW7	North-West Slope	42°05'28.5612"N	127°42'41.9652"E	898	Alfisols	Temperate deciduous broad-leaved forest
NW8	North-West Slope	42°02'54.4092"N	127°40'26.0796"E	888	Alfisols	Temperate deciduous broad-leaved forest
S1	South Slope	41°58'17.6628"N	128°03'51.0840"E	2374	Cambisols	Alpine tundra
S2	South Slope	41°56'8.9304"N	128°4'31.5012"E	1983	Cambisols	Alpine tundra
S3	South Slope	41°54'5.9940"N	128°05'15.9468"E	1749	Alfisols	Cold temperate and temperate mountain coniferous forests
S4	South Slope	41°51'1.6308"N	128°05'56.9436"E	1467	Andosols	Cold temperate and temperate mountain coniferous forests
S5	South Slope	41°47'29.6268"N	128°06'7.9164"E	1374	Alfisols	Cold temperate and temperate mountain coniferous forests

Table S1. The classification of potential ecological risk.

Er^i			RI		
Classification	Hakanson	This research	Classification	Hakanson	This research
Slight	<40	<40	Slight	<150	<40
Moderate	40-80	40-80	Moderate	150-300	40-80
Considerable	80-160	80-160	Considerable	300-600	80-160
High	160-320	160-320	Very high	≥600	≥160
Very high	≥320	≥320			

Table S2. Parameter values used in health assessment.

Parameter	Description	Unit	Value	
			Children	Adult
C	Concentration of metal	$mg \cdot kg^{-1}$		
EF	Exposure frequency	$d \cdot year^{-1}$	350	350
ED	Exposure duration	year	6	30
BW	Body weight	kg	15	70
AT	Averaging time	d	ED × 365	ED × 365
IngR	Soil ingestion rate	$mg \cdot d^{-1}$	200	100
InhR	Soil inhalation rate	$m^3 \cdot d^{-1}$	7.6	20
PEF	Particulate emission factor	$m^3 \cdot kg^{-1}$	1.36×10^9	1.36×10^9
SA	Skin surface area	$cm^2 \cdot d^{-1}$	5700	2800
SAF	Skin adherence factor	$mg \cdot cm^{-2}$	0.2	0.07
ABS_d	Dermal absorption factor	unitless	0.001	0.001
RfD_{ing}	Ingestion reference dose	$mg \cdot kg^{-1} \cdot d^{-1}$	As(0.0003), Co(0.02), Cd(0.001), Cr(0.003), Mn(0.046) ,Ni(0.02), Pb(0.0035), Zn(0.3), Cu(0.04)	
RfD_{inh}	Inhalation reference dose	$mg \cdot kg^{-1} \cdot d^{-1}$	As(5×10^{-5}), Co(5.71×10^{-6}), Cd(5.7×10^{-6}), Cr(2.86×10^{-5}), Mn(1.43×10^{-5}), Ni(0.0206), Pb(3.52×10^{-3}), Zn(0.3), Cu(0.0402)	
RfD_{dermal}	Dermal reference dose	$mg \cdot kg^{-1} \cdot d^{-1}$	As(0.0003), Co(0.016), Cd(1×10^{-5}), Cr(6×10^{-5}), Mn(0.14), Ni(0.0054), Pb(5.25×10^{-4}), Zn(0.06), Cu(0.012)	
SF_{ing}	Ingestion slope factor	$kg \cdot d^{-1} \cdot mg^{-1}$	As(1.5), Cd(6.3), Cr(0.5), Ni(0.84)	
SF_{inh}	Inhalation slope factor	$kg \cdot d^{-1} \cdot mg^{-1}$	As(15.1), Cd(6.3), Cr(42), Ni(0.84)	
SF_{dermal}	Dermal slope factor	$kg \cdot d^{-1} \cdot mg^{-1}$	As(3.66)	

Table S3. Tianchi volcano and global distribution of metals in volcanic soil and volcanic ash.

Metal concentrations (mg kg^{-1})	Volcanic soil							Volcanic ash					Background values of soil elements in China [11]
	Tianchi volcano, China	Fernando de Noronha, Brazilian [1]	Vesuvius National Park, Italy [2]	Wusu Tianshan volcano, China [3]	Wudalianchi volcano, China [4]	Santiago Island, Cape Verde [5]	Popocatépetl volcano, Mexico [6]	Tianchi volcano, China	Popocatepetl volcano, Mexico [7]	Cordón Caulle, Chile [8]	Puna, Argentina [9]	Mt. Etna volcano, Italy [10]	
Al	6966.77	-	54410.88	18237.35	-	-	-	5464.88	69400	2442.38	-	168000	59500
Fe	14939.2	-	29584	50678.57	-	-	-	15973.3	35700	4663.5	-	77900	27400
K	18663.4	-	41931.5	16958.66	-	-	-	27470.4	8300	165.63	-	33400	19400
Ca	2201.0	-	38767	4472.2	-	-	-	1892.42	44400	1890.63	-	10800	12600
Na	17496.9	-	10023.88	14912.01	-	-	-	34967.8	30700	709.25	-	59800	15400
Mg	3498.0	-	10396	8779.43	-	-	-	497.12	23300	660.13	-	53100	6800
Mn	451.79	-	883.75	1239.13	-	-	-	518.62	685	48.75	594.57	1420	636
Ti	4230.36	-	1489.5	50.84	-	-	-	2161.26	3100	-	-	-	4100
Cu	5.78	24.01	186.63	36.18	17.59	51.78	9-140	0.9505	26.66	22	7.7	106	17.1
Pb	9.43	<LD	91.73	16.86	10.55	5.94	45-77	16.16	9.16	4.75	31.21	<2	28.8
Zn	162.16	97.48	112.75	84.03	44.12	83.6	58-98	366.97	74	10.375	59.39	160	80.4
Cr	22.63	237.7	5.57	69.14	31.69	135.18	52-159	0.8236	62	-	11.3	24.9	46.7
Ni	7.28	45.81	24.54	34.3	18.54	137.46	50-152	0.1498	46.91	0.9125	6.4	-	21.4
Ba	221.71	522.15	823.75	183.41	-	-	-	24.32	338	-	274.73	-	529
Ga	30.72	-	-	17.09	-	-	-	171.53	16.33	-	20.28	-	16.6
Li	27.77	-	-	39.64	-	-	-	40.66	17.33	-	70.2	-	29.7
Co	4.59	13.02	-	17.78	-	45.93	-	1.95	17.16	2.875	15.3	-	11.9
Cd	<LD	<LD	0.49	0.28	-	0.21	4-6	<LD	0.57	-	-	<1	0.099
As	8.04	<LD	16.93	21.87	5.96	0.05	0.31-2.2	3.0982	-	1.15	5.2	132	8
Sn	2.93	-	-	-	-	-	-	6.76	-	-	3.37	-	2.7
Sr	19.97	-	-	30.94	-	-	-	3.72	515.25	-	143.76	-	187

-not determined, <LD below the detection limit

Table S4. Pearson's correlation coefficient between metals in volcanic soil and volcanic ash.

	Al	Fe	K	Ca	Na	Mg	Mn	Ti	Cu	Pb	Zn	Cr	Ni	Ba	Ga	Li	Co	As	Sn	Sr
Al	1																			
Fe	0.010	1																		
K	-0.486**	0.542**																		
Ca	0.299	-0.015	-0.411*	1																
Na	-0.358	0.542**	0.894**	-0.339	1															
Mg	0.503**	-0.255	-0.759**	0.364	-0.890**	1														
Mn	0.093	0.548**	0.259	-0.006	0.405*	-0.284	1													
Ti	0.489**	-0.170	-0.577**	0.342	-0.735**	0.862**	-0.199	1												
Cu	0.500**	-0.162	-0.662**	0.249	-0.711**	0.826**	-0.067	0.649**	1											
Pb	-0.661**	0.487**	0.805**	-0.292	0.702**	-0.641**	0.142	-0.625**	-0.492**	1										
Zn	-0.518**	0.609**	0.831**	-0.230	0.865**	-0.798**	0.340	-0.733**	-0.605**	0.804**	1									
Cr	0.434*	-0.338	-0.750**	0.348	-0.914**	0.967**	-0.344	0.891**	0.771**	-0.632**	-0.828**	1								
Ni	0.458*	-0.211	-0.769**	0.375*	-0.894**	0.972**	-0.195	0.835**	0.843**	-0.624**	-0.765**	0.951**	1							
Ba	0.503**	-0.387*	-0.753**	0.276	-0.885**	0.909**	-0.280	0.872**	0.777**	-0.760**	-0.808**	0.908**	0.907**	1						
Ga	0.383*	-0.260	-0.493**	0.258	-0.581**	0.618**	-0.238	0.618**	0.511**	-0.545**	-0.584**	0.627**	0.578**	0.651**	1					
Li	-0.165	0.423*	0.598**	-0.237	0.516**	-0.292	0.048	-0.261	-0.109	0.493**	0.616**	-0.329	-0.267	-0.259	-0.270	1				
Co	0.480**	-0.278	-0.673**	0.274	-0.798**	0.874**	-0.107	0.899**	0.740**	-0.644**	-0.735**	0.896**	0.875**	0.885**	0.592**	-0.237	1			
As	0.334	0.496**	0.074	-0.070	0.066	0.148	0.448*	0.261	0.209	-0.142	-0.050	0.079	0.179	0.178	0.221	-0.048	0.118	1		
Sn	-0.475**	0.453*	0.801**	-0.231	0.804**	-0.710**	0.109	-0.696**	-0.543**	0.892**	0.870**	-0.724**	-0.713**	-0.817**	-0.576**	0.661**	-0.682**	-0.244	1	
Sr	0.572**	-0.416*	-0.765**	0.395*	-0.869**	0.923**	-0.279	0.883**	0.760**	-0.751**	-0.843**	0.915**	0.873**	0.926**	0.618**	-0.346	0.878**	0.074	-0.768**	1

*Significant at 0.05 level, **Significant at 0.01 level.

References

1. Fabricio Neta, A.B.; do Nascimento, C.W.A.; Biondi, C.M.; van Straaten, P.; Bittar, S.M.B. Natural concentrations and reference values for trace elements in soils of a tropical volcanic archipelago. *Environ. Geochem. Health* **2018**, *40*, 163–173, doi:10.1007/s10653-016-9890-5.
2. Memoli, V.; Eymar, E.; García-Delgado, C.; Esposito, F.; Santorufo, L.; Marco, A.D.; Barile, R.; Maisto, G. Total and fraction content of elements in volcanic soil: Natural or anthropogenic derivation. *Sci. Total Environ.* **2018**, *625*, 16–26, doi:10.1016/j.scitotenv.2017.12.223.
3. Jiang, Y.; Yin, Z.; Zhang, Y.; Wang, C.; Cao, J. Qualitative and quantitative analysis of metals and detection of colony in volcano mud. *China Surfactant Deterg. Cosmet.* **2016**, *46*, 729–732, doi:10.13218 /j.cnki.csdc.2016.12.011.
4. Wang, X.; Fang, Z.; Wu, J.; Cao, Y. The analysis of heavy metal pollution investigation of Wudalianchi volcano mud. *Heilongjiang Sci.* **2016**, *7*, 37–39.

5. Cabral Pinto, M.M.S.; Ferreira da Silva, E.; Silva, M.M.V.G.; Melo-Gonçalves, P. Heavy metals of Santiago Island (Cape Verde) top soils: Estimated Background Value maps and environmental risk assessment. *J. Afr. Earth Sci.* **2015**, *101*, 162–176, doi:10.1016/j.jafrearsci.2014.09.011.
6. Rodriguez-Espinosa, P.F.; Jonathan, M.P.; Morales-García, S.S.; Villegas, L.E.C.; Martínez-Tavera, E.; Muñoz-Sevilla, N.P.; Cardona, M.A. Metal enrichment of soils following the April 2012–2013 eruptive activity of the Popocatépetl volcano, Puebla, Mexico. *Environ. Monit. Assess.* **2015**, *187*, 717, doi:10.1007/s10661-015-4938-z.
7. Shruti, V.C.; Rodríguez-Espinosa, P.F.; Martinez-Tavera, E.; Hernández-Gonzalez, D. Metal concentrations in recent ash fall of Popocatepetl volcano 2016, Central Mexico: Is human health at risk? *Ecotoxicol. Environ. Saf.* **2018**, *162*, 324–333, doi:10.1016/j.ecoenv.2018.06.067.
8. Stewart, C.; Craig, H.M.; Gaw, S.; Wilson, T.; Villarosa, G.; Outes, V.; Cronin, S.; Oze, C. Fate and agricultural consequences of leachable elements added to the environment from the 2011 Cordón Caulle tephra fall. *J. Volcanol. Geotherm. Res.* **2016**, *327*, 554–570, doi:10.1016/j.jvolgeores.2016.09.017.
9. Ruggieri, F.; Saavedra, J.; Fernandez-Turiel, J.L.; Gimeno, D.; Garcia-Valles, M. Environmental geochemistry of ancient volcanic ashes. *J. Hazard. Mater.* **2010**, *183*, 353–365, doi:10.1016/j.jhazmat.2010.07.032.
10. Toscano, G.; Caristi, C.; Cimino, G.; Sorption of heavy metal from aqueous solution by volcanic ash. *C. R. Chim.* **2008**, *11*, 765–771, doi:10.1016/j.crci.2007.11.010.
11. CNEMC (The Chinese Environmental Monitoring Centre). *The Background Values of Soil Elements in China*; Chinese Environment Science Press: Beijing, China, 1990.
